# Unveiling the glycolysis in sepsis: Integrated bioinformatics and machine learning analysis identifies crucial roles for *IER3*, *DSC2*, and *PPARG* in disease pathogenesis

**DOI:** 10.1097/MD.0000000000039867

**Published:** 2024-09-27

**Authors:** Dongqing Cui, Tian Yu

**Affiliations:** a Emergency Department, Beijing Sixth Hospital, Beijing, China; b Chinese Academy of Medical Sciences and Peking Union Medical College, Beijing, China.

**Keywords:** glycolytic signaling, immune response, machine learning, sepsis

## Abstract

Sepsis, a multifaceted syndrome driven by an imbalanced host response to infection, remains a significant medical challenge. At its core lies the pivotal role of glycolysis, orchestrating immune responses especially in severe sepsis. The intertwined dynamics between glycolysis, sepsis, and immunity, however, have gaps in knowledge with several Crucial genes still shrouded in ambiguity. We harvested transcriptomic profiles from the peripheral blood of 107 septic patients juxtaposed against 29 healthy controls. Delving into this dataset, differential expression analysis shed light on genes distinctly linked to glycolysis in both cohorts. Harnessing the prowess of LASSO regression and SVM-RFE, we isolated Crucial genes, paving the way for a sepsis risk prediction model, subsequently vetted via Calibration and decision curve analysis. Using the CIBERSORT algorithm, we further mapped 22 immune cell subtypes within the septic samples, establishing potential interactions with the delineated Crucial genes. Our efforts unveiled 21 genes intricately tied to glycolysis that exhibited differential expression patterns. Gene set enrichment analysis (GSEA) and Kyoto Encyclopedia of Genes and Genomes (KEGG) pathway analyses offered insights, spotlighting pathways predominantly associated with oxidative phosphorylation, PPAR signaling pathway, Glycolysis/Gluconeogenesis and HIF-1 signaling pathway. Among the myriad genes, *IER3*, *DSC2*, and *PPARG* emerged as linchpins, their prominence in sepsis further validated through ROC analytics. These sentinel genes demonstrated profound affiliations with various immune cell facets, bridging the complex terrain of glycolysis, sepsis, and immune responses. In line with our endeavor to “unveil the glycolysis in sepsis,” the discovery of *IER3*, *DSC2*, and *PPARG* reinforces their cardinal roles in sepsis pathogenesis. These revelations accentuate the intricate dance between glycolysis and immunological shifts in septic conditions, offering novel avenues for therapeutic interventions.

## 1. Introduction

Sepsis, often termed a “silent killer,” has continued to be a major global health concern, with estimates suggesting nearly 48.9 million cases and 11 million related deaths annually.^[1,2]^ Despite advances in medical science, the mortality rates from sepsis remain disconcertingly high, emphasizing the urgent need for improved diagnostic and therapeutic strategies.^[2]^ Underlying the clinical presentations of sepsis is a highly complex biochemical and immunological interplay, with glycolysis at its epicenter.

Glycolysis, a fundamental metabolic pathway, has traditionally been associated with energy generation. However, its expanded role in immune cell function, especially under pathological conditions like sepsis, is emerging.^[3]^ Glycolytic metabolism fuels immune responses by catering to the bioenergetic and biosynthetic demands of activated immune cells.^[4,5]^ A dysregulated glycolysis, therefore, can potentially tilt the immune balance, driving the host response from controlled defense to overwhelming inflammation, characteristic of sepsis.

Though the symbiotic relationship between sepsis and glycolysis is recognized, the exact molecular mechanisms, specifically the Crucial genes that mediate this relationship, remain enigmatic. While certain genes have been previously associated with the septic response, such as *PFKFB3*, which modulates glycolysis, a bifunctional enzyme that intimately tie glycolysis to sepsis has yet to be achieved.^[6]^

Given this backdrop, the present study leverages advanced bioinformatics and machine learning tools to shed light on this gray area. By parsing through transcriptomic profiles of septic patients and employing state-of-the-art analytical algorithms, we aim to unmask pivotal genes orchestrating the nexus between glycolysis and sepsis.

## 2. Materials and methods

### 2.1. Transcriptomic data acquisition and differential gene analysis related to glycolysis in sepsis

Peripheral blood mRNA expression profiles of sepsis patients were sourced from Gene Expression Omnibus datasets GSE131761^[7]^ and GSE154918.^[8]^ GSE131761 contained blood samples from 81 sepsis patients and 15 healthy controls, while GSE154918 incorporated 26 sepsis patients alongside 14 controls.^[7,8]^ To merge and adjust for batch effects between these datasets, the ComBat function of the sva package was utilized.^[9]^ This resulted in a consolidated dataset encompassing 107 sepsis patients and 29 healthy controls for subsequent analyses.

A curated list of 353 glycolysis-related genes was retrieved from the Molecular Signature Database (https://www.gsea-msigdb.org/gsea/msigdb) (see TableS1, Supplemental Digital Content, http://links.lww.com/MD/N652). Differential gene expression in sepsis was determined using the limma package in R with thresholds set at |Log2FC| > 1 and a false discovery rate (FDR) < 0.05.^[10]^ Genes meeting these criteria were cross-referenced with the glycolysis-related genes to pinpoint glycolysis-associated differential genes in sepsis.

### 2.2. Functional and pathway enrichment analysis

The clusterprofiler^[11]^ R package was employed for gene set enrichment analysis (GSEA).^[12]^ GSEA evaluated the distribution of genes from predefined sets in a ranked gene list correlated to phenotype, thus determining the contribution to the phenotype.^[12]^ The “c2.cp.v7.5.1.symbols.gmt” gene set, extracted from the Molecular Signatures Database (MSigDB) database (v7.5.1), was utilized for GSEA, with significance set at *P* value < .05.^[13]^ Furthermore, the clusterProfiler package was used to facilitate Gene Ontology (GO) functional annotation and Kyoto Encyclopedia of Genes and Genomes (KEGG) pathway enrichment analyses for glycolysis-related differential genes.^[14,15]^ Significance was anchored at *P* value < .05.

### 2.3. Machine learning-based crucial gene selection

To identify potential crucial genes, we employed 2 advanced machine learning techniques: Least Absolute Shrinkage and Selection Operator (LASSO) regression and Support Vector Machine (SVM) with Recursive Feature Elimination (RFE). LASSO regression, known for its regularization and feature selection capabilities, was utilized to enhance predictive accuracy and identify genes significantly associated with sepsis. This analysis was performed using the “glmnet” package in R.^[16]^ The glmnet function was applied to our dataset, where a binomial family was specified to account for the binary nature of our outcome (sepsis patients vs healthy individuals). Through this process, genes with non-zero coefficients were identified, indicating their significant distinction between the 2 groups. The selection was visualized and fine-tuned by plotting lambda values against log(lambda), allowing for the identification of the optimal lambda that minimizes cross-validation error. SVM, a widely used supervised learning algorithm for classification and regression tasks, was applied in conjunction with RFE, a technique for feature selection that recursively removes the least important features. To adapt SVM for multi-class classification and enhance feature selection, we utilized the mSVM-RFE algorithm, integrating multi-class SVM with RFE, following the methodology detailed by Duan et al.^[17]^ This approach was particularly efficient for handling our extensive feature set by optionally pruning half the features per iteration, significantly reducing computational complexity without compromising the model’s ability to identify crucial genes. To ensure the robustness and reliability of our selected gene features, model accuracy and feature selection efficacy were evaluated using 10-fold cross-validation. This method involves partitioning the dataset into ten subsamples, using 9 for training and 1 for validation iteratively. This approach allows for a comprehensive assessment of the model’s performance and its generalizability to unseen data.

### 2.4. Immune infiltration analysis

To estimate the relative abundance of 22 immune cell types in the samples, CIBERSORTx, an advanced computational method designed for deriving cell type proportions from gene expression data in complex tissues, was applied.^[18]^ A signature matrix of 22 immune cell types, comprising gene expression signatures of 22 distinct immune cell types, was selected.^[19]^ Relative abundance results for the 22 immune cell types were procured, discarding immune cells with compositions of zero in over half the samples.

### 2.5. Logistic regression modeling and nomogram visualization

In our statistical modeling, a logistic regression framework was established using the rms package. To visually elucidate the predictive model, a nomogram was constructed. We then evaluated the model’s robustness through a bootstrap-based calibration, ensuring a consistent alignment between predicted and observed outcomes. To further determine the clinical significance and utility of our identified gene markers, we employed decision curve analysis using the rmda package.^[20]^ The resultant curves provided pivotal visual data, archived for subsequent reference. Furthermore, the translational potential of our model was accentuated by the generation of a clinical impact curve, highlighting its tangible applicability in clinical contexts.

### 2.6. Statistical analysis

In our analysis, all computational and statistical evaluations were executed using R software (version 4.2), focusing on the assessment of continuous variables between sepsis patients and healthy controls. To accommodate the diverse distribution characteristics of our data, we employed the Independent Student *t* test for normally distributed variables, ensuring comparisons were made on the basis of mean differences. For data not adhering to normality, the Mann–Whitney *U* test served as our non-parametric alternative, focusing on median differences without assuming data distribution. Both tests were conducted as 2-tailed, with a predetermined significance level of *P* < .05, to capture potential differences in either direction without bias. In addressing the challenge of multiple comparisons, *P* values were adjusted using the FDR method to control for type I errors, striking a balance between the detection of genuine effects and the minimization of false positives.

## 3. Results

### 3.1. Glycolytic gene dysregulation marks the onset of sepsis

**Figure 1.**
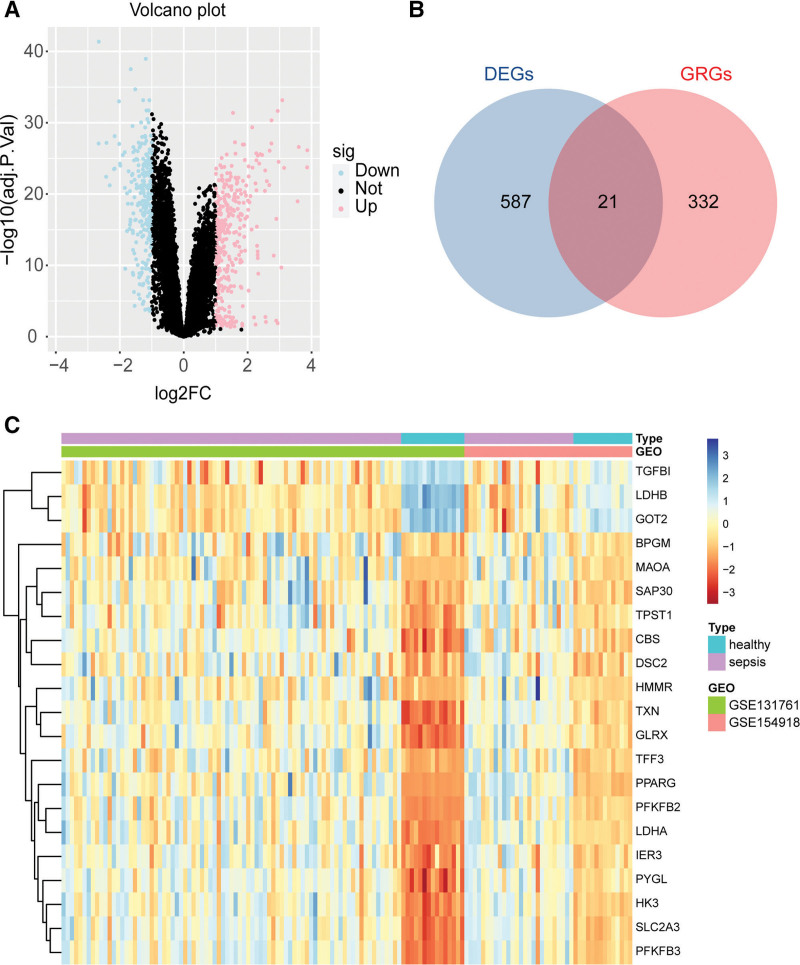
Differential expression of glycolysis-related genes between sepsis patients and controls. (A) Volcano plot displays the distribution of genes based on their log2 fold change and significance (−log10(adj.p.val)), highlighting downregulated (blue), upregulated (pink), and non-significant (black) genes. (B) Through the Venn diagram, an overlap of 21 genes is discerned between differentially expressed genes (DEGs) shown in blue and glycolysis-related genes (GRGs) represented in pink. (C) Heatmap visualizes gene expression patterns, with color gradients from blue (low) to red (high), alongside sample types (healthy or sepsis) and gene datasets (GSE131761, GSE154918). DEG = differentially expressed genes, GRG = glycolysis-related genes.

Our investigations commenced with a comprehensive gene expression analysis between sepsis patients and controls (Fig. 1). Interestingly, a subset of genes involved in glycolysis displayed marked differences in their expression profiles. Based on the threshold set for differentially expressed genes, 354 genes were upregulated and 254 genes were downregulated in the sepsis cohort (Fig. 1A). The volcano plot (Fig. 1A) crystallized the gene expression landscape, with certain glycolysis-related genes being prominently upregulated or downregulated Intriguingly, a convergence of 21 genes was discerned between the Differentially Expressed Genes and the glycolysis-related genes, suggesting a tight-knit association between glycolysis and sepsis pathogenesis (Fig. 1B). We further visualized the expression patterns of these 21 genes across sepsis and control groups using a heatmap (Fig. 1C).

### 3.2. Sepsis triggers broad metabolic and cellular shifts

**Figure 2.**
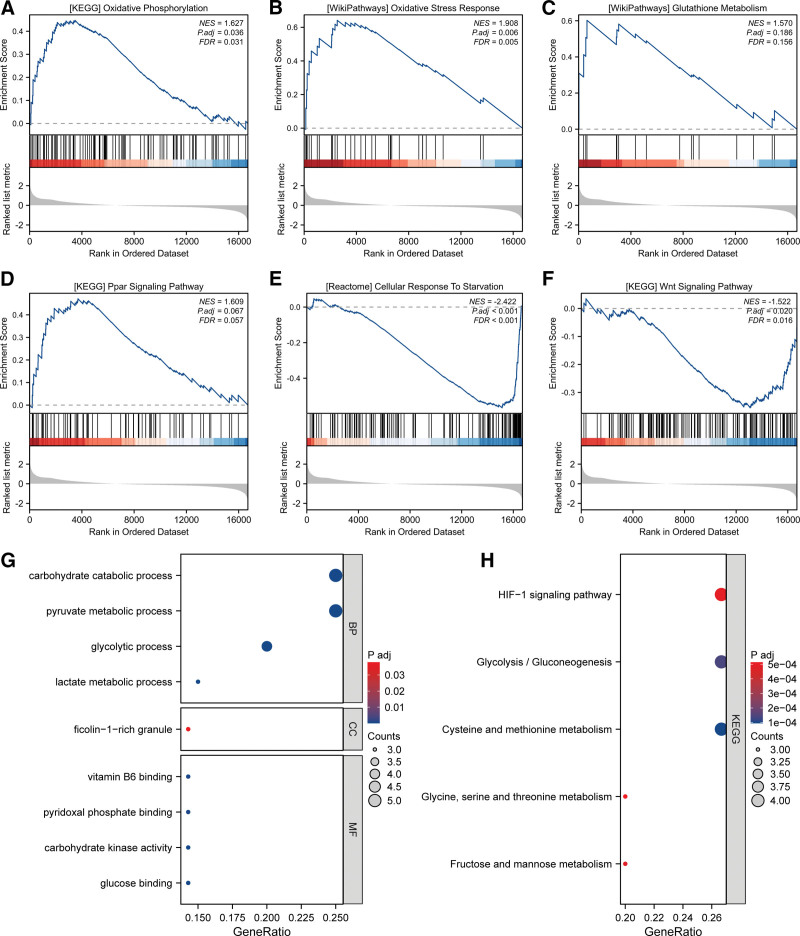
Pathway enrichment analyses highlighting distinct metabolic and cellular responses in sepsis compared to healthy controls. (A–F) Gene set enrichment analysis (GSEA) conducted on all genes between sepsis patients and healthy controls, revealing various pathways: (A) KEGG “Oxidative Phosphorylation” (NES = 1.627, FDR = 0.031), (B) WikiPathways “Oxidative Stress Response” (NES = 1.096, FDR = 0.065), (C) WikiPathways “Glutathione Metabolism” (NES = 1.570, FDR = 0.156), (D) KEGG “PPAR Signaling Pathway” (NES = −0.634, FDR = 0.057), (E) Reactome “Cellular Response to Stress” (NES = −2.422, FDR < 0.001), (F) KEGG “Wnt Signaling Pathway” (NES = −1.522, FDR = 0.016). (G) Gene Ontology (GO) analysis represented as a dot plot, illustrating various biological processes such as carbohydrate catabolism and glycolytic activity. The size of each dot indicates gene count, while the color gradient denotes the adjusted *P* value (P adj). (H) KEGG pathway analysis depicted as a dot plot, emphasizing pathways including HIF-1 signaling and glycolysis/gluconeogenesis. Each dot’s color gradient signifies the adjusted *P* value (P adj), and the size represents the number of genes associated with each pathway.

To delve deeper, we explored overarching metabolic routes and cellular shifts instigated by sepsis (Fig. 2). A GSEA sweep illuminated pathways such as “Oxidative Phosphorylation,” “PPAR signaling pathway,” and others, highlighting an intensified metabolic adjustment in sepsis sufferers (Figs. 2A–F). An integrative interpretation reveals an enhanced metabolic response rooted in oxidative stress and cellular adaptation to nutritional paucity. Furthermore, the dot plot from the Gene Ontology and KEGG pathway evaluations (Figs. 2G–H) emphasized the disrupted cellular and metabolic activities, implying a wholesale metabolic transformation in sepsis. Specifically, GO was enriched in processes like pyruvate metabolic process and carbohydrate catabolic process (Fig. 2G), while KEGG underscored pathways such as “Glycolysis/Gluconeogenesis” and “HIF-1 signaling pathway” (Fig. 2H). These results collectively spotlight the metabolic and cellular overhaul that sepsis induces, emphasizing its profound systemic impacts.

### 3.3. Deciphering key molecular signatures of sepsis

**Figure 3.**
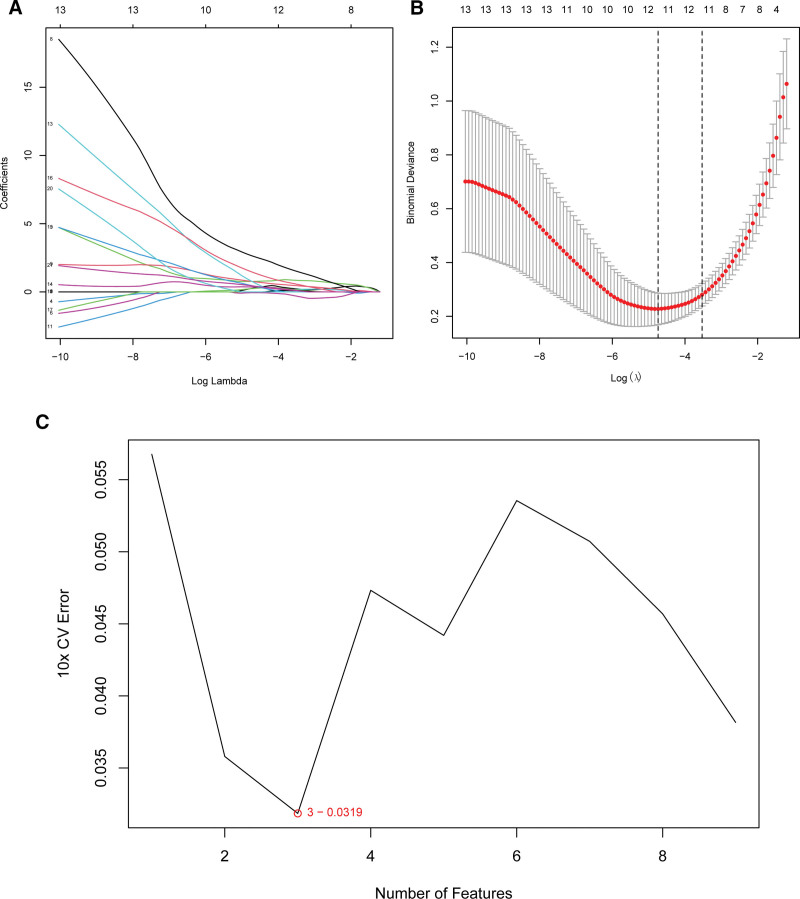
Feature selection and model evaluation for identifying key glycolysis-related genes in sepsis. (A) Lasso regression analysis: Lasso regression identified 11 candidate genes with non-zero coefficients (highlighted in color) as potential contributors to sepsis-related glycolysis. The coefficient trajectories were calculated across a range of log(λ) values. (B) Model performance in Lasso regression: The binomial deviance plot illustrates the model’s performance during Lasso regression. The red curve represents the average binomial deviance, while specific lambda values associated with non-zero coefficients are marked for potential gene selection. (C) SVM-RFE for gene selection: support vector machine-recursive feature elimination (SVM-RFE) analysis revealed that an optimal subset of 3 genes (highlighted in color) contributed significantly to glycolysis in sepsis. The 10-fold cross-validation error reached a minimum value of 0.0319 at this feature count.

**Figure 4.**
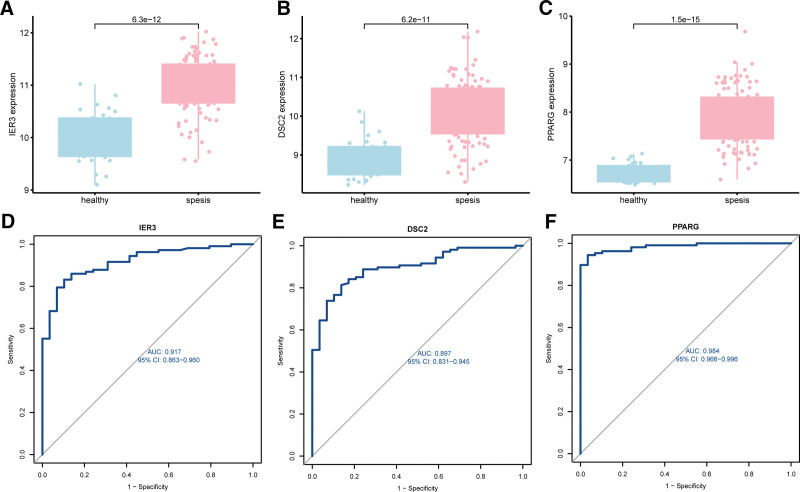
Differential expression of crucial genes in peripheral blood between sepsis patients and healthy controls, and their receiver operating characteristic (ROC) curves. (A–C) Box plots depict the expression levels of the genes *IER3* (A), *DSC2* (B), and *PPARG* (C) in healthy individuals (blue) compared to sepsis patients (pink). Each dot represents an individual sample, and the *P* values indicate significant differences in gene expression between the 2 groups. (D–F) ROC curves illustrate the diagnostic performance of the genes *IER3* (D), *DSC2* (E), and *PPARG* (F) in distinguishing sepsis from healthy controls. The area under the curve (AUC) values and their 95% confidence intervals (CIs) are provided for each gene. The diagonal gray line represents the line of no discrimination. AUC = area under the curve, CI = confidence interval, ROC = receiver operating characteristic.

In our quest to uncover the central molecular drivers, we identified a series of genes linked with glycolysis perturbations in sepsis (Fig. 3). Harnessing the power of sophisticated statistical methodologies such as Lasso Regression and SVM-RFE, we delineated a subset of genes that may underpin the glycolytic shift characteristic of sepsis. Notably, through LASSO, the genes SLC2A3, TXN, GOT2, PPARG, CBS, GLRX, IER3, TFF3, DSC2, HMMR, and BPGM emerged as significant (Figs. 3A,B). Delving deeper with SVM-RFE, we spotlighted 3 genes: IER3, DSC2, and PPARG, positing a pivotal role in the glycolytic dynamics during septic progression (Fig. 3C). A comparative expression analysis between sepsis patients and healthy controls revealed a marked upregulation of these 3 genes in sepsis subjects (Figs. 4A–C). To quantify their diagnostic potency, we charted ROC curves for each gene, yielding the following insights:For IER3, DSC2, and PPARG, the AUC values were 0.917 (95% CI: 0.863–0.960), 0.897 (95% CI: 0.831–0.945), and 0.984 (95% CI: 0.966–0.996) respectively (Figs. 4D–F).

### 3.4. The immune landscape in sepsis revealed

**Figure 5.**
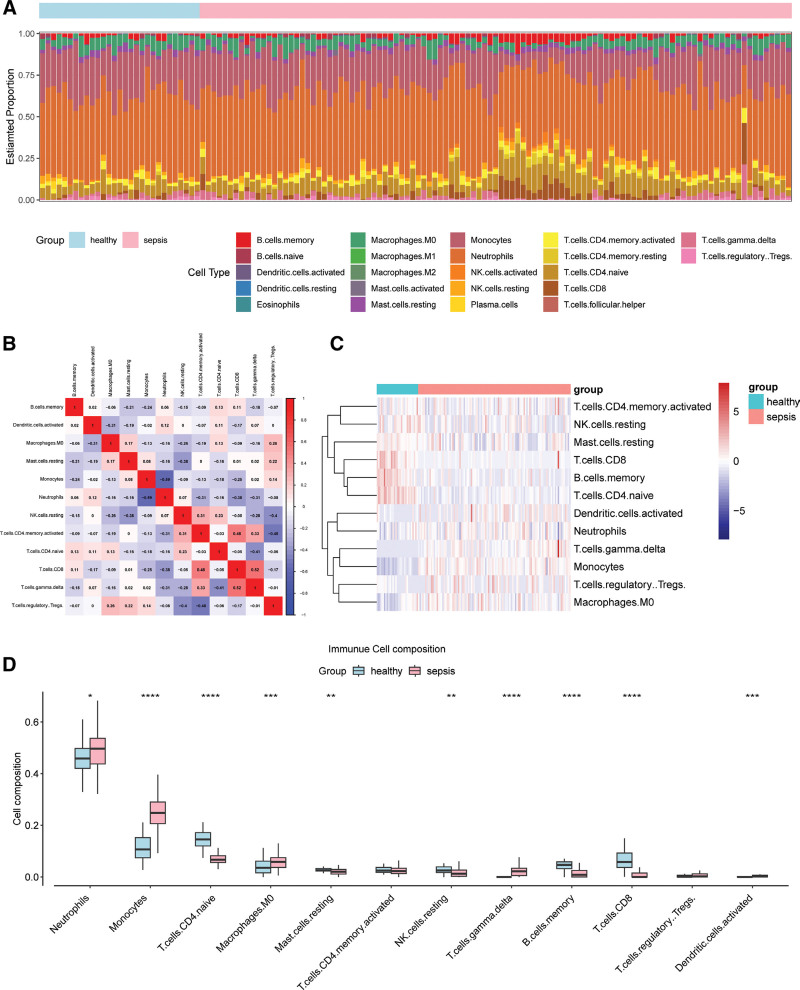
Comparative analysis of immune cell composition between sepsis patients and healthy controls. (A) Stacked bar plot illustrating the estimated proportion of various immune cell types in healthy individuals (left) and sepsis patients (right). Each bar represents an individual sample, and colors indicate different cell types as shown in the legend below. (B) Heatmap displaying the correlation matrix between immune cell types. Positive correlations are shown in blue, and negative correlations in red. The intensity of the color and accompanying number represent the strength of the correlation. (C) Hierarchical clustering of immune cell types based on their expression profiles in both groups. The heatmap reflects the z-score normalized expression of each cell type, with blue indicating lower expression and red indicating higher expression. The side color bar denotes the group: healthy (blue) and sepsis (red). (D) Box plots comparing the cell composition of select immune cells between healthy (blue) and sepsis (red) groups. The Y-axis represents the proportion of each cell type. Significance levels are denoted as: *P < .05, **P < .01, ***P < .001, and ****P < .0001.

In our investigation of the nexus between metabolic reprogramming and immune responses, we turned our attention to the immune landscape characteristic of sepsis (Fig. 5A). Sepsis patients exhibited a starkly different immune cell composition, hinting at a perturbed immune response (Fig. 5A). Intriguing correlations between immune cell types further revealed a complex interplay, possibly driven by the altered metabolic state (Figs. 5B,C). Upon examining the immune cell composition, sepsis patients exhibited pronounced elevations in innate immune cells, including neutrophils, monocytes, M0 macrophages, and activated dendritic cells. Additionally, there was a noticeable increase in the specific adaptive immune cell type, γδ T-cells. In stark contrast, sepsis patients demonstrated reduced populations in the adaptive immunity sector, characterized by a decline in naive CD4 T-cells, CD8 T-cells, and memory B cells. Furthermore, a decrease was evident in resting states of innate immune cells, notably mast cells and NK cells (Fig. 5D).

### 3.5. Linking gene expression to immune dysregulation

**Figure 6.**
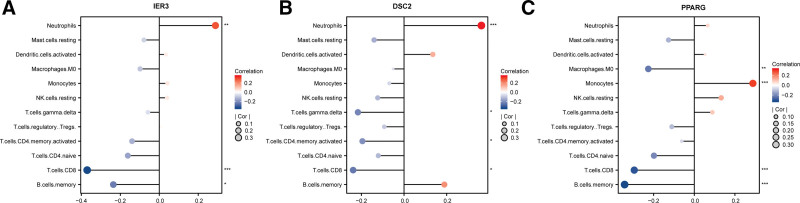
Associations between crucial gene expressions and immune cell distributions in sepsis patients. (A) Dot plots depicting the correlation coefficients between the expression of IER3 and immune cell types. IER3 demonstrates a significant positive correlation with neutrophils. Conversely, a negative association is observed with CD8+ T cells and B cells memory. (B) Scatter plots illustrating the correlation strengths between DSC2 gene expression and immune cell subsets. DSC2 exhibits a notable positive correlation with neutrophils and a negative correlation with activated memory CD4+ T cells, gamma delta T cells, and CD8+ T cells. (C) The correlation matrix illustrates the relationship between *PPARG* gene expression and various immune cell populations. Specifically, there’s a positive correlation between PPARG and monocytes, whereas a negative correlation is observed with resting mast cells, CD8+ T cells, memory B cells, and M0 Macrophages. Each dot in the matrix indicates the magnitude and direction of the correlation for a particular immune cell type in relation to the gene expression.

In our endeavor to unravel the connection between gene expression and immune function, a pivotal focus was directed towards relationships between key glycolytic genes and a spectrum of immune cell types (Figs. 6A–C). A noteworthy observation was the pronounced correlation between the expression of IER3 and neutrophils (Fig. 6A). This gene, which plays a role in cellular responses to environmental stress, showcased a significant positive correlation with neutrophils. Conversely, a diminished association was evident with CD8+ T cells and memory B cells, suggesting that IER3 might have distinct roles in modulating different immune cell functions. In contrast, the DSC2 gene, implicated in cell-cell adhesion, demonstrated varying correlations across immune cell types. It aligned positively with neutrophils, but this was juxtaposed by its negative associations with activated memory CD4+ T cells, gamma delta T cells, and CD8+ T cells(Fig. 6B). Lastly, the interplay of PPARG, a gene pivotal in lipid metabolism and inflammation, with various immune cells, offered further insights. It revealed a robust positive correlation with monocytes, implying a potential role in monocyte activation or recruitment in sepsis. However, the gene’s negative correlations with resting mast cells, CD8+ T cells, memory B cells, and M0 Macrophages suggest that its modulatory effects on immune responses in sepsis might be expansive and diverse(Fig. 6C).

### 3.6. Establishing a predictive sepsis paradigm with glycolytic gene signatures

**Figure 7.**
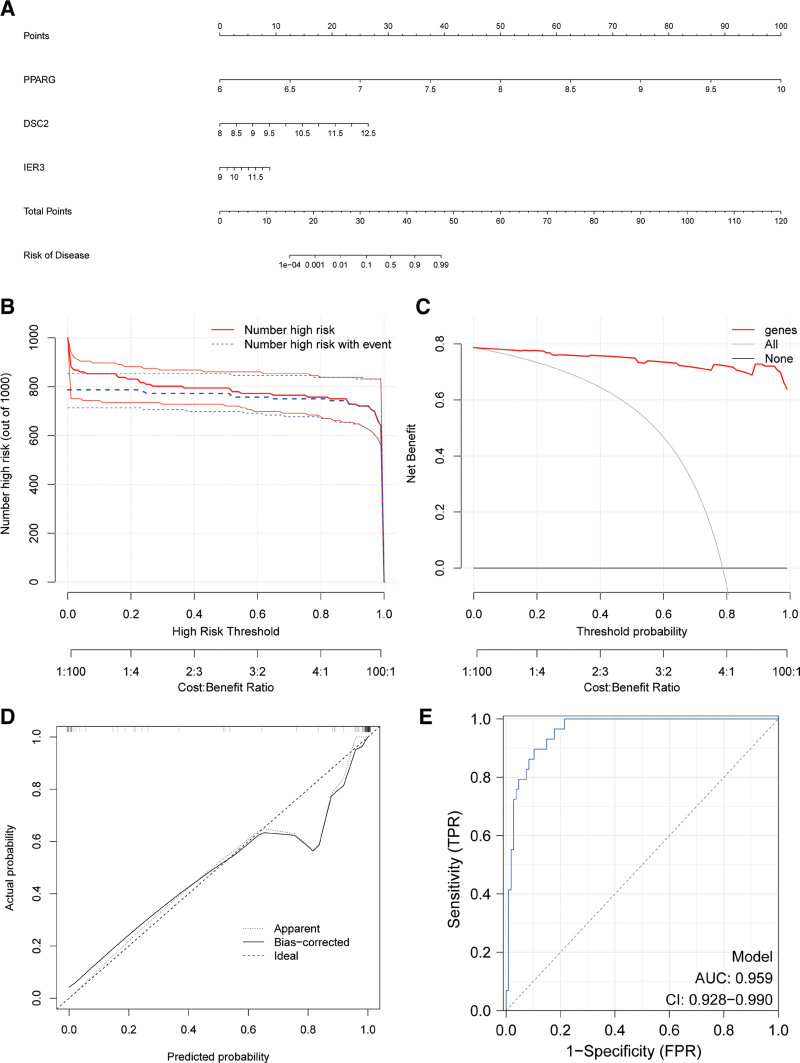
Analysis of sepsis prediction based on the expression of 3 key glycolysis genes. (A) Nomogram representation: Correlation of the expression levels of 3 key glycolysis genes, namely *PPARG*, *DSC2*, and *IER3*, with the risk of sepsis. The bottom total points represent the cumulative scores, which indicate the potential risk of disease. (B) High-risk analysis: The solid red line tracks the number of individuals identified as high-risk for sepsis, while the dotted blue line reveals those within the high-risk category who experienced an event, mapped against different high-risk thresholds. (C) Net benefit analysis: The visual representation showcases the net benefit associated with including (genes, red line) or excluding (none, blue line) all genes in the predictive model. The decision curve assesses the advantages of utilizing this predictive model across various threshold probabilities. (D) Calibration plot: This plot outlines the relationship between predicted and actual probabilities of sepsis. The dashed line is the ideal prediction, the solid line depicts the bias-corrected probability, and the dotted line displays the apparent probability. (E) ROC curve analysis: The receiver operating characteristic (ROC) curve illustrates model sensitivity against 1-specificity for sepsis prediction. With an area under the curve (AUC) of 0.959 and a confidence interval (CI) ranging from 0.928 to 0.990, the robustness of the predictive model is evident. AUC = area under the curve, CI = confidence interval, ROC = receiver operating characteristic.

Harnessing the expression profiles of pivotal glycolytic genes: PPARG, DSC2, and IER3: we’ve devised a predictive model that seeks to revolutionize the diagnostic approach to sepsis. The nomogram serves as a tangible manifestation of how gene expressions correlate with sepsis susceptibility, potentially acting as a game-changer in early clinical interventions (Fig. 7A). Further strengthening the model’s validity, the High-Risk Analysis delineates the subsets at amplified risk, with a congruence between predicted vulnerabilities and actual septic events (Fig. 7B). The Net Benefit Analysis decisively swings the balance in favor of our gene-centric approach, emphasizing its clinical utility (Fig. 7C). This notion finds resonance in the Calibration Plot where predicted probabilities align closely with actual sepsis outcomes (Fig. 7D). Culminating our findings, the ROC Curve Analysis delivers an AUC of 0.959, ensconced within a confidence interval of 0.928 to 0.990, showcasing the model’s robustness (Fig. 7E).

## 4. Discussion

Sepsis remains a perplexing dilemma in the realm of clinical medicine, consistently pushing the boundaries of our existing therapeutic strategies.^[21,22]^ Central to its complex pathophysiological network is glycolysis: a metabolic transition that is notably intensified under septic conditions.^[23–25]^ This study delves deeply into this intricate interplay, bringing to light novel genes profoundly connected to the glycolytic pathway within the context of sepsis. Our high-throughput transcriptomic profiling, involving 107 septic patients and 29 healthy controls, elucidated 21 glycolysis-associated genes, thereby enriching the sepsis-glycolysis paradigm. Of these, IER3, DSC2, and PPARG stand out as paramount for their roles in septic pathophysiology.

We observed a significant upregulation of IER3 expression in the peripheral blood of septic patients. The multifactorial inducement of this gene, spanning growth factors, cytokines, ionizing radiation, viral infections, and other cellular stresses, underscores its pivotal role in response to a diverse array of stimuli.^[26]^ Previous work by Sina et al^[27]^ provided crucial insights into the influence of IER3 in the realm of immunological responses. Through the utilization of ier3 knockout mice, they delved into the ramifications of ier3 absence on intestinal inflammatory reactions.^[27]^ In their experiments, an experimental model of colitis induced by oral dextran sulfate sodium (DSS) highlighted a stark reality: the loss of the ier3 gene profoundly exacerbated both acute and chronic colonic inflammation.^[27]^ As the DSS-treated mice manifested increased disease phenotypes, the functional deprivation of ier3 bolstered monocyte reactivity, especially in terms of NF-κB activation and pro-inflammatory molecule release.^[27]^ Of equal significance is the revelation from earlier studies on inflammatory bowel disease (IBD). Costello et al^[28]^ identified IER3 as one of the most upregulated genes in patients with ulcerative colitis and Crohn’s disease. In addition, research by Sven Flemming and colleagues discovered that mice with an inducible conditional knockdown of Dsc2 exhibited compromised mucosal repair following biopsy-induced colonic injury and recovery from dextran sulfate sodium-induced colitis. The knockdown of Dsc2 was linked to a decrease in cell-matrix traction forces, reduced levels of integrin β1 and β4, and alterations in the activity of the small GTPase Rap1.^[29]^

While our findings build on this knowledge, they pave the way for further exploration, particularly in evaluating the therapeutic potential of modulating IER3 in conditions like sepsis and IBD. In the study by DPhil and colleagues, a cohort of 265 sepsis patients and 106 controls was analyzed to map eQTLs, shedding light on the genetic intricacies of sepsis.^[30]^ Among the findings, the IER3 was identified as a regulatory genetic variant, emphasizing its potential role in sepsis response.^[30]^ This association not only highlights the complex genetic landscape of sepsis but also suggests potential avenues for therapeutic exploration.

Sepsis is characterized by the substantial production of inflammatory mediators.^[31]^ Central to this process is the peroxisome proliferator-activated receptor γ (PPARγ), a ligand-dependent transcription factor from the nuclear receptor superfamily, which is integral in directing inflammatory pathways.^[32]^ Its expression is noted across a broad range of immune cells, including neutrophils, lymphocytes, and macrophages.^[33,34]^ Existing literature underscores PPARγ’s role in neutrophil migration, macrophage phagocytosis, and the modulation of inflammatory markers.^[33]^ Notably, those with PPARG gene mutations might be at an increased risk for exacerbated inflammatory responses and sepsis, with associated poorer outcomes.^[35]^ The rs10865710 polymorphism in the enhancer region may further impact transcription factor binding, subsequently influencing PPARγ expression and vulnerability to traumatic septicemia.^[36]^ As a result, both PPARγ and its associated ligands have been identified as promising treatment avenues for sepsis and various inflammatory conditions.

Given recent revelations about the pivotal role of immune cell infiltration in sepsis pathogenesis, our data, highlighting the positive correlation of IER3 and DSC2 with neutrophils and their negative association with CD8+ T cells, as well as the inverse relationship of PPARG with monocytes, offers valuable insights into potential immune cell-gene interactions in the septic milieu. In our exploration, we identify a clear linkage between key glycolytic genes, IER3 and DSC2, and the behavior of neutrophils during sepsis. It’s been documented that, in the context of sepsis, hampering glycolysis results in neutrophil immunosuppression.^[37]^ This phenomenon may be steered by the PI3K/Akt-HIF-1α pathway-mediated LDHA downregulation.^[37]^ A notable contribution by Sadiku et al^[38]^ highlights that neutrophils capitalize on the glycogen cycle for essential energy production, a critical determinant for their function and survival. Disruptions in this metabolic framework have implications for chronic disease manifestations. Our findings further enrich the understanding of neutrophil metabolic profiles in the throes of acute inflammation, with a specific lens on glycolysis.

While our study has provided valuable insights into the relationship between glycolysis and sepsis, there are inherent limitations. Firstly, our sample size, consisting of 107 septic patients and 29 controls, though robust, may not capture the complete variability present in the general population. Additionally, while our high-throughput transcriptomic profiling has spotlighted key glycolytic genes such as IER3, DSC2, and PPARG, further validation in diverse cohorts is necessary. The focus on peripheral blood expression may not represent the full systemic complexity of sepsis, and a broader investigation into other tissues could be revealing. Lastly, our observations provide correlations but do not necessarily confirm causative relationships, warranting deeper mechanistic studies.

Peering into the future, these discoveries on IER3, DSC2, and PPARG chart avenues for targeted therapeutic strategies, possibly encompassing gene therapy or tailored immunomodulation: aligning with the paradigm of precision medicine. Furthermore, probing the interplay between these genes and other metabolic pathways could unveil deeper immunoregulatory mechanisms.

## 5. Conclusion

In conclusion, our exploration underscores the pivotal role of glycolysis in sepsis, emphasizing the significance of IER3, DSC2, and PPARG. By shedding light on these molecular protagonists, we contribute to a more comprehensive understanding of sepsis, providing a springboard for future research and potential therapeutic innovations. Advancing this knowledge will bolster efforts against this global health menace, potentially reducing its alarming mortality and morbidity rates.

## Author contributions

**Conceptualization:** Dongqing Cui, Tian Yu.

**Data curation:** Dongqing Cui.

**Formal analysis:** Dongqing Cui, Tian Yu.

**Methodology:** Dongqing Cui, Tian Yu.

**Software:** Dongqing Cui.

**Supervision:** Dongqing Cui.

**Visualization:** Tian Yu.

**Writing – original draft:** Dongqing Cui, Tian Yu.

**Writing – review & editing:** Dongqing Cui.

## Supplementary Material


